# Supplementary material for: Altered sulfation status of FAM20C-dependent chondroitin sulfate is associated with osteosclerotic bone dysplasia
Source: Nat Commun. 2022 Dec 26;13:7952. doi: 10.1038/s41467-022-35687-3 (PMC9792594; doi:10.1038/s41467-022-35687-3)
Supplement: Supplementary file 1 — Supplementary Information [file 41467_2022_35687_MOESM1_ESM.pdf]

**Supplementary Information**

# Altered sulfation status of FAM20C-dependent chondroitin sulfate is associated with osteosclerotic bone dysplasia.

**Toshiyasu Koike[1], Tadahisa Mikami[1], Jun-ichi Tamura[2] & Hiroshi Kitagawa[1]**

[1]Laboratory of Biochemistry, Kobe Pharmaceutical University, Higashinada-ku, Kobe 658-8558, Japan. [2]Department of Agricultural, Life and Environmental Sciences, Faculty of Agriculture, Tottori University, Tottori 680-8551, Japan.

Correspondence to: Hiroshi Kitagawa (kitagawa@kobepharma-u.ac.jp)

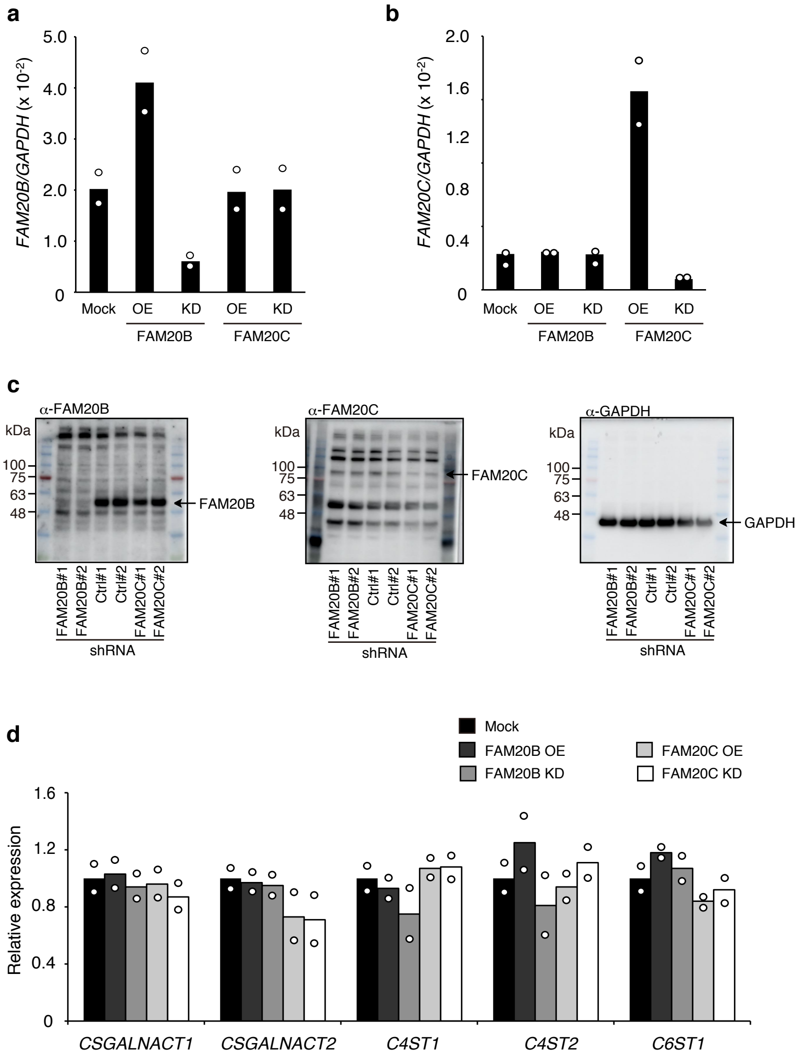


**Supplementary Figure 1** Establishment of FAM20B-related or FAM20C-related stable HeLa cell lines. (**a**,**b**) Relative expression level of the transcripts for FAM20B (**a**) or FAM20C (**b**) (normalized to that of *GAPDH*) in mock-transfected (Mock), FAM20B overexpressing (OE), FAM20B knockdown (KD), FAM20C OE, and FAM20C KD HeLa cell lines (n =2 independent experiments). (**c**) Cell lysates from FAM20B KD (FAM20B#1 and #2), non-targeting control (Ctrl#1 and #2), and FAM20C KD (FAM20C#1 and #2) HeLa cell lines were analyzed by immunoblotting to evaluate knockdown efficiency for FAM20B and FAM20C. GAPDH was used as a loading control. Data are obtained from three independent experiments and representative images are shown. (**d**) Expression profiles of genes involved in CS biosynthesis in stable HeLa cell lines. The expression level of the respective transcripts was normalized to that of *GAPDH* (n = 2 independent experiments). Source data are provided as a Source Data file.

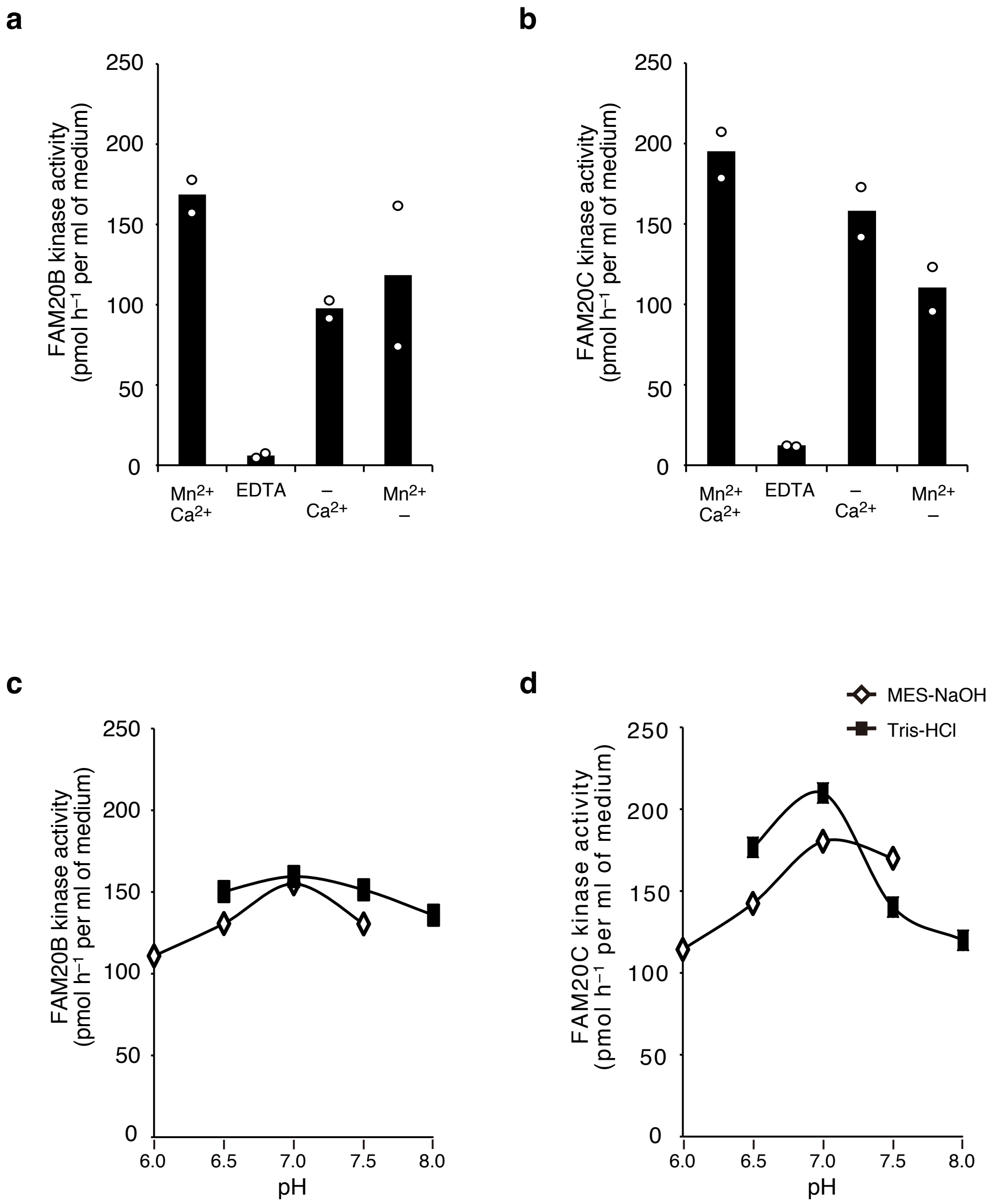


**Supplementary Figure 2** Enzymatic characteristics of soluble forms of FAM20B and FAM20C. The metal ion dependence (**a**,**b**) and the pH dependence (**c**,**d**) for Xyl kinase activities of FAM20B (**a**,**c**) and FAM20C (**b**,**d**) toward GlcAβ1-3Galβ1-3Galβ1-4Xylβ1-*O*-SerGlyTrpProAspGly were determined under standard assay conditions in the presence of divalent cations or EDTA in different buffers. Data in **a** and **b** are the mean values from two independent experiments. Data in **c** and **d** correspond to raw data from single experiment. Source data are provided as a Source Data file.

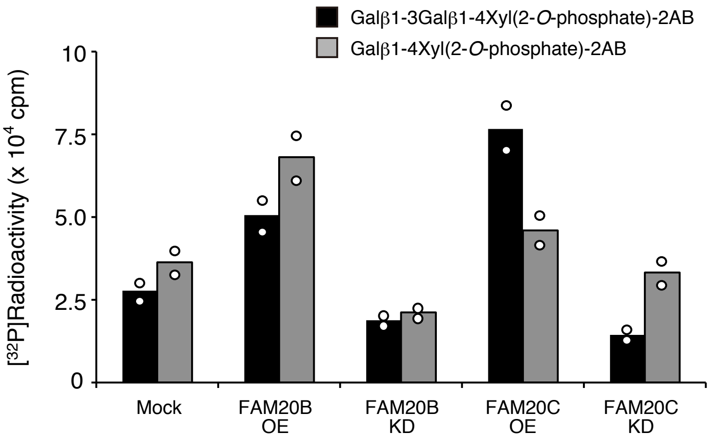


**Supplementary Figure 3** Detection of the GAG linkage saccharide intermediates from FAM20B-related or FAM20C-related stable HeLa cell lines. Data are the mean values from two independent experiments. Source data are provided as a Source Data file.

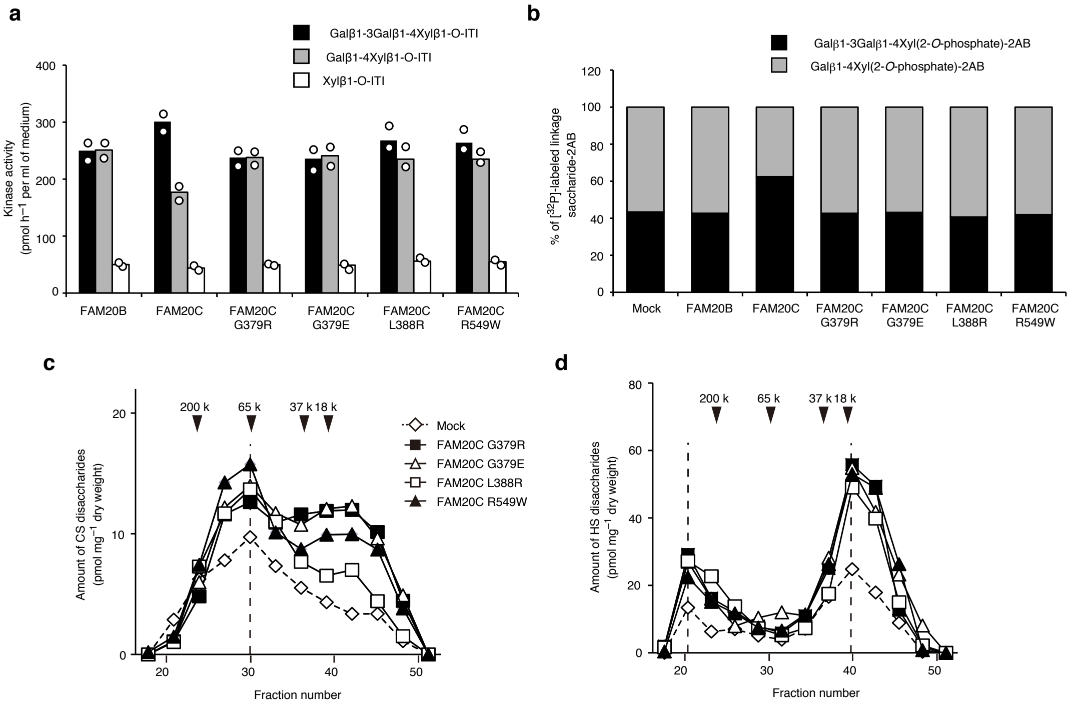


**Supplementary Figure 4** XYLK activity-dependent GAG biosynthesis is not affected by Raine syndrome mutations in FAM20C. (**a**) Kinase activities of soluble forms of FAM20 proteins toward ITI preparations. Data are the mean values from two independent experiments. (**b**) The relative proportions of the linkage saccharide intermediates from stable HeLa cell lines overexpressing wild-type FAM20B, wild-type FAM20C or mutant FAM20C proteins. Data are the mean values from two independent experiments. (**c**, **d**) Gel filtration elution profiles of (**c**) CS and (**d**) HS chains from stable HeLa cell lines overexpressing each of the mutant FAM20C proteins. Numbered arrowheads (200 k, 65 k, 37 k, and 18 k) indicate the elution position of 200-, 65-, 37-, and 18-kDa saccharides derived from size-defined commercial dextran, respectively. The profile data for mock cells in **c** and **d** are identical to those in Figs. 1**d** and **e**, respectively. Results represent the average of two series of independent experiments. Source data are provided as a Source Data file.

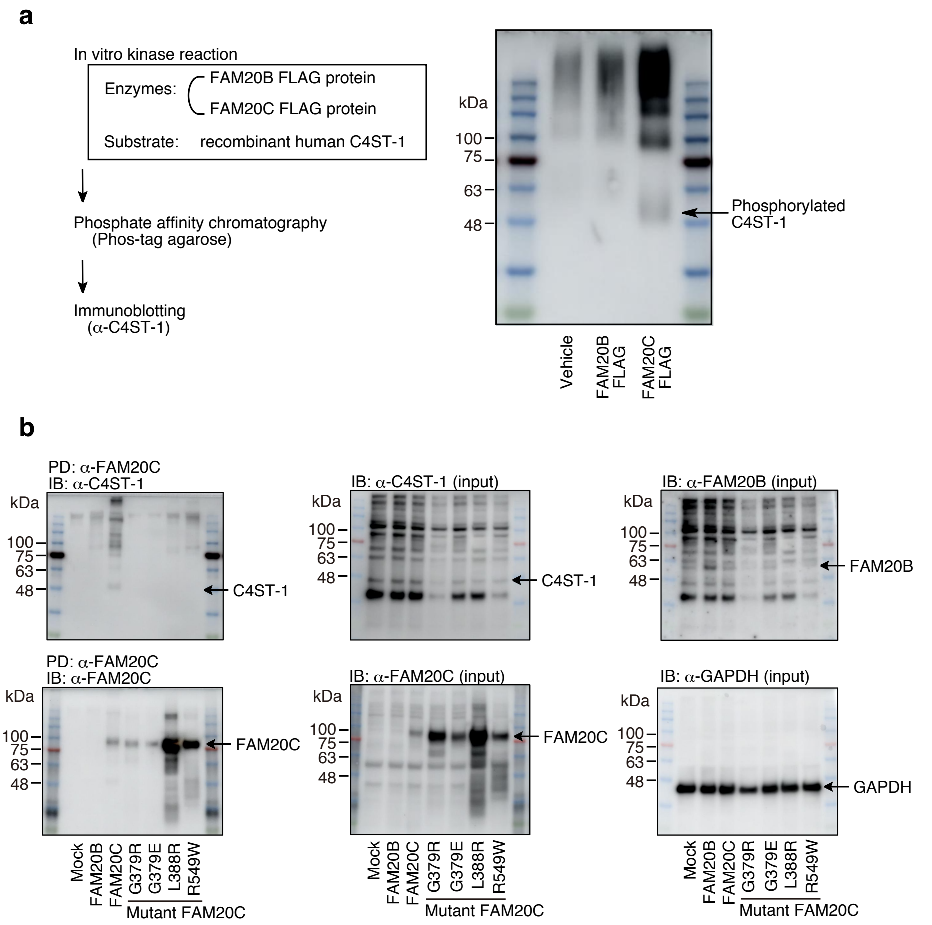


**Supplementary Figure 5** FAM20C interacts with C4ST-1. (**a**) Recombinant C4ST-1 can be phosphorylated by a soluble form of FLAG-tagged FAM20C, but not that of FAM20B, *in vitro*. An *in vitro* kinase assay was performed by incubating recombinant C4ST-1 and the purified FLAG-tagged FAM20 proteins. The phosphorylated proteins in the reaction mixtures were isolated by phosphate affinity chromatography using phos-tag agarose. The resultant samples were analyzed by immunoblotting using an anti-C4ST-1 antibody (L18). Data are obtained from three independent experiments and a representative image is shown. (**b**) Wild-type FAM20C, but not its Raine syndrome mutants, can physically interact with endogenous C4ST-1 in Saos-2 cells. The anti-FAM20C immunoprecipitates from formaldehyde-treated cell lysates of stable Saos cell lines overexpressing FAM20 proteins were analyzed by immunoblotting using anti-C4ST-1 antibody (L18) and anti-FAM20C antibody. Data are obtained from three independent experiments and representative images are shown. Source data are provided as a Source Data file.

**a**

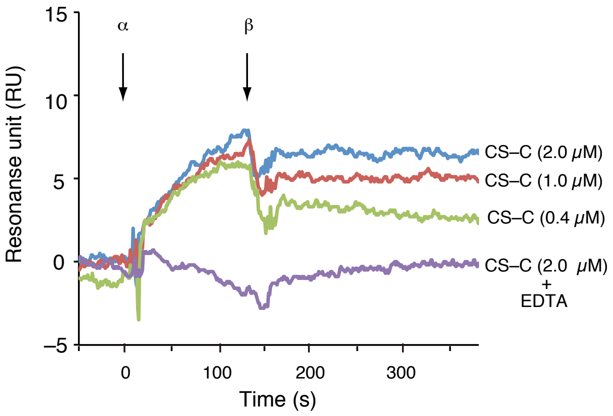


**b**

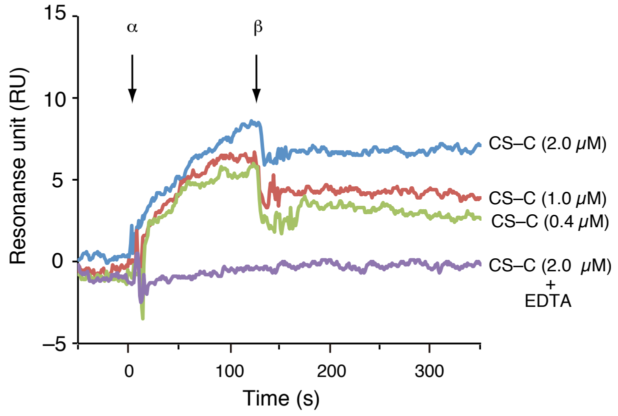


**c**

| GAG vs protein | *ka* | *kd* | $K_d$ |
|---|---|---|---|
| | $M^{-1}$ $S^{-1}$ | $S^{-1}$ | nM |
| CS–C vs N-cadherin | $4.0 \times 10^3$ | $6.8 \times 10^{-4}$ | $1.7 \times 10^2$ |
| CS–C vs Cadherin-11 | $3.6 \times 10^3$ | $6.9 \times 10^{-4}$ | $1.9 \times 10^2$ |

**Supplementary Figure 6** CS-C binds to N-cadherin and cadherin-11. (**a**,**b**) Overlaid sensorgrams for the binding of CS-C to immobilized N-cadherin (**a**) or cadherin-11 (**b**) in a BIAcore system. Various concentrations of CS-C were individually injected over the N-cadherin- or cadherin-11-immobilized sensor chip in the presence of 3 mM $CaCl_2$ or 3 mM EDTA. The beginning of the association and dissociation phases is marked by arrows labeled α and β, respectively. RU, resonance units. (**c**) Kinetic parameters for the interaction of CS-C with immobilized N-cadherin or cadherin-11. Values of the apparent association (*ka*) and dissociation (*kd*) rate constants, and equilibrium dissociation constants (*Kd*), are expressed as mean ± s.d. Source data are provided as a Source Data file.

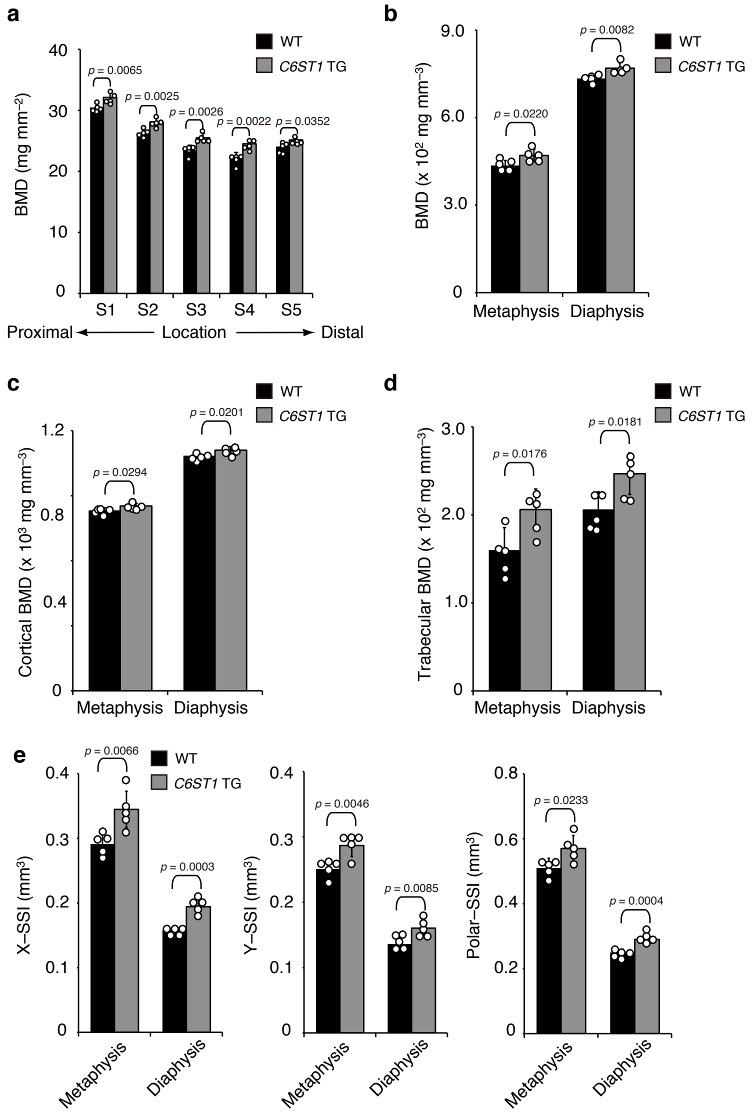


**Supplementary Figure 7** Assessment of the BMD of tibias from 16-week-old wild-type (WT) or *C6ST1* transgenic (TG) male mice. (**a**) BMD of each of 5 equal longitudinal divisions of the tibias were determined by peripheral quantitative computed tomography, pQCT (n = 5 bones from total, each from different litters, unpaired Student's *t* test, two-sided). (**b-d**) Dual energy X-ray absorptiometry (DXA)-derived measurements of BMD at the tibial metaphysis and diaphysis (n = 5 bones from total, each from different litters, unpaired Student's *t* test, two-sided). (**e**) X-axis, Y-axis, and polar stress-strain indexes (SSIs) of tibias from 16-week-old WT or *C6ST1* TG male mice were calculated as surrogate measurements of bone mechanical strength (n = 5 bones from total, each from different litters, unpaired Student's *t* test, two-sided). Data in **a-e** are represented as mean ± s.d. Source data are provided as a Source Data file.

**Supplementary Table 1** Disaccharide composition of GAG chains from stable clones of HeLa cells.

| | pmol $mg^{-1}$ (mol%)[a] | | | | | | |
|---|---|---|---|---|---|---|---|
| CS disaccharides | FAM20C KD | Mock | FAM20C OE | FAM20C G379R | FAM20C G379E | FAM20C L388R | FAM20C R549W |
| ΔHexA[b]-GalNAc | 1.1 ± 1.0 (3) | 1.7 ± 0.3 (3) | 1.7 ± 0.5 (2) | 2.1 ± 0.6 (3) | 3.5 ± 0.4 (4) | 1.1 ± 0.1 (2) | 3.1 ± 2.4 (3) |
| ΔHexA-GalNAc(6S) | 7.0 ± 0.8 (20) | 8.7 ± 1.5 (17) | 9.5 ± 1.1 (10) | 15.3 ± 1.0 (18) | 16.6 ± 0.8 (18) | 14.3 ± 1.2 (20) | 17.8 ± 1.7 (20) |
| ΔHexA-GalNAc(4S) | 24.9 ± 0.6 (71) | 37.9 ± 4.0 (74) | 73.0 ± 11.0 (79) | 61.1 ± 4.0 (73) | 66.2 ± 3.7 (72) | 52.4 ± 2.3 (73) | 65.2 ± 5.7 (72) |
| ΔHexA(2S)-GalNAc(6S) | 0.7 ± 0.3 (2) | 1.2 ± 0.3 (2) | 0.7 ± 0.6 (1) | 1.8 ± 0.2 (2) | 2.0 ± 0.2 (2) | 1.5 ± 0.5 (2) | 1.6 ± 0.3 (2) |
| ΔHexA-GalNAc(4S,6S) | 1.1 ± 0.4 (3) | 1.9 ± 0.7 (4) | 7.5 ± 1.6 (8) | 3.5 ± 0.4 (4) | 3.6 ± 0.4 (4) | 2.6 ± 0.5 (4) | 3.1 ± 0.3 (3) |
| Total CS disaccharides | 34.9 ± 1.8 (100) | 51.4 ± 3.7 (100) | 92.4 ± 9.7 (100) | 83.7 ± 3.7 (100) | 91.9 ± 2.3 (100) | 71.8 ± 4.2 (100) | 91.0 ± 5.4 (100) |
| HS disaccharides | FAM20C KD | Mock | FAM20C OE | FAM20C G379R | FAM20C G379E | FAM20C L388R | FAM20C R549W |
| ΔHexA-GlcNAc | 33.6 ± 2.2 (48) | 49.4 ± 5.7 (44) | 101.1 ± 2.9 (50) | 111.3 ± 4.1 (49) | 116.6 ± 9.0 (48) | 92.6 ± 2.2 (47) | 112.1 ± 5.0 (52) |
| ΔHexA-GlcNAc(6S) | 0.7 ± 0.4 (1) | 1.8 ± 0.5 (2) | 2.3 ± 0.7 (1) | 2.3 ± 0.7 (1) | 2.4 ± 0.4 (1) | 2.0 ± 0.2 (1) | 2.3 ± 0.6 (1) |
| ΔHexA-GlcN(NS) | 20.3 ± 6.1 (29) | 36.2 ± 6.0 (32) | 72.2 ± 1.7 (36) | 79.5 ± 12.1 (35) | 87.5 ± 9.4 (36) | 69.0 ± 2.8 (35) | 81.8 ± 4.6 (38) |
| ΔHexA-GlcN(NS,6S) | 0.8 ± 0.4 (1) | 2.5 ± 0.8 (2) | 4.5 ± 0.9 (2) | 4.5 ± 1.2 (2) | 4.9 ± 0.3 (2) | 3.9 ± 0.2 (2) | 4.7 ± 1.1 (2) |
| ΔHexA(2S)-GlcN(NS) | 7.7 ± 3.6 (11) | 10.9 ± 1.3 (10) | 13.8 ± 7.7 (7) | 18.2 ± 1.8 (8) | 19.4 ± 2.3 (8) | 15.8 ± 0.2 (8) | 18.7 ± 1.5 (8) |
| ΔHexA(2S)-GlcN(6S,NS) | 6.9 ± 3.7 (10) | 12.2 ± 1.8 (11) | 9.7 ± 1.1 (5) | 11.4 ± 2.3 (5) | 12.2 ± 0.7 (5) | 13.8 ± 1.9 (7) | 14.0 ± 1.7 (6) |
| Total HS disaccharides | 69.9 ± 9.9 (100) | 113.0 ± 13.3 (100) | 203.6 ± 13.2 (100) | 227.2 ± 18.3 (100) | 243.0 ± 19.8 (100) | 197.0 ± 5.3 (100) | 233.6 ± 14.1 (100) |
| Relative expression[c] | 0.3 | 1.0 | 5.4 | 9.4 | 10.9 | 4.5 | 9.5 |

[a]The values are expressed as pmol of disaccharide per mg of dried homogenate and represent the mean ± s.d. (n = 3).

[b]ΔHexA, GalNAc, GlcNAc, and GlcN represent unsaturated hexuronic acid, *N*-acetylgalactosamine, *N*-acetylglucosamine, and glucosamine, respectively; 6S, 4S, 2S, and NS represent 6-*O*-sulfate, 4-*O*-sulfate, 2-*O*-sulfate, and 2-*N*-sulfate, respectively.

[c]Quantitative real-time RT-PCR was used to measure relative amounts of the *FAM20C* transcript. *FAM20C* mRNA levels were normalized to that of *GAPDH* (glyceraldehyde-3-phosphate dehydrogenase).

Source data are provided as a Source Data file.

**Supplementary Table 2** Comparison of kinase activities of fusion proteins secreted into the culture medium by the transfected COS-1 cells.

| Acceptor substrate | FAM20B | FAM20C | FAM20B(D309G) | FAM20B(D478G) |
|---|---|---|---|---|
| | Kinase activity (pmol $h^{-1}$ per ml of medium)[a] | | | |
| α-TM[b] | 171.2 ± 17.6 | 188.6 ± 18.1 | –[c] | – |
| GlcAβ1-3Galβ1-3Galβ1-4Xylβ1-*O*-SerGlyTrpProAspGly | 155.6 ± 2.9 | 178.2 ± 46.1 | ND[d] | ND |
| Galβ1-3Galβ1-4Xylβ1-*O*-Ser | 50.2 ± 3.5 | 57.7 ± 14.0 | – | – |
| Galβ1-3Galβ1-4Xyl(2-*O*-phosphate)β1-*O*-Ser | ND | ND | – | – |

[a]The values are the mean ± s.d. For FAM20B and FAM20C, n = 3; For FAM20B(D309G) and FAM20C(D478G), n = 2.

[b]α-TM contains a tetrasaccharide linkage, GlcAβ1-3Galβ1-3Galβ1-4Xyl[58].

[c]–, not tested.

[d]ND, not detected ( < 0.1 pmol $h^{-1}$ per ml of medium).

Source data are provided as a Source Data file.

**Supplementary Table 3** Comparison of kinase activities of FAM20B and FAM20C toward ITI preparations.

| Acceptor substrate | FAM20B | FAM20C |
|---|---|---|
| | Kinase activity (pmol $h^{-1}$ per ml of medium) [a] | |
| Galβ1-3Galβ1-4Xylβ1-*O*-ITI | 249.8 ± 33.0 | 298.6 ± 18.3 |
| Galβ1-4Xylβ1-*O*-ITI | 251.2 ± 32.4 | 176.7 ± 21.4 |
| Xylβ1-*O*-ITI | 49.9 ± 11.6 | 44.1 ± 8.9 |

[a]The values are the mean ± s.d. (n = 3).

Source data are provided as a Source Data file.

**Supplementary Table 4** Disaccharide composition of CS chains from stable clones of murine fibroblastic L and its mutant cell line, sog9 cells.

| | pmol mg$^{-1}$ (mol%)[a] | | | | |
|---|---|---|---|---|---|
| CS disaccharides | L-mock | L-FAM20B-1 | L-FAM20B-2 | L-FAM20C-1 | L-FAM20B-2 |
| ΔHexA-GalNAc | 36.9 ± 0.7 (10) | 45.2 ± 2.8 (9) | 48.6 ± 6.7 (10) | 35.0 ± 2.8 (8) | 33.5 ± 0.7 (6) |
| ΔHexA-GalNAc(6S) | 11.2 ± 0.2 (3) | 18.1 ± 1.3 (4) | 22.8 ± 3.7 (5) | 10.9 ± 2.7 (2) | 11.9 ± 2.7 (2) |
| ΔHexA-GalNAc(4S) | 275.5 ± 1.7 (76) | 365.3 ± 13.4 (76) | 374.3 ± 46.7 (74) | 348.8 ± 16.8 (78) | 442.5 ± 14.5 (80) |
| ΔHexA(2S)-GalNAc(6S) | ND[b] | ND | ND | ND | ND |
| ΔHexA-GalNAc(4S,6S) | 41.1 ± 1.0 (11) | 54.9 ± 4.1 (11) | 57.6 ± 7.9 (11) | 51.1 ± 3.0 (11) | 65.2 ± 1.3 (12) |
| Total CS disaccharides | 364.8 ± 1.1 (100) | 483.5 ± 21.4 (100) | 503.3 ± 64.9 (100) | 445.7 ± 19.0 (100) | 553.1 ± 14.7 (100) |
| Relative expression of *FAM20B*[c] | 1.0 | 2.0 | 3.5 | 1.0 | 1.1 |
| Relative expression of *FAM20C*[c] | 1.0 | 1.2 | 1.0 | 2.1 | 3.6 |
| CS disaccharides | sog9-mock | sog9-FAM20B-1 | sog9-FAM20B-2 | sog9-FAM20C-1 | sog9-FAM20B-2 |
| ΔHexA-GalNAc | 44.2 ± 2.0 (50) | 67.4 ± 2.7 (60) | 73.8 ± 7.1 (60) | 76.3 ± 2.5 (62) | 81.4 ± 7.1 (63) |
| ΔHexA-GalNAc(6S) | 15.2 ± 0.3 (17) | 21.0 ± 1.7 (19) | 23.9 ± 1.4 (19) | 22.2 ± 1.9 (18) | 24.1 ± 0.6 (19) |
| ΔHexA-GalNAc(4S) | 27.9 ± 1.0 (32) | 23.9 ± 0.4 (21) | 24.4 ± 2.8 (20) | 23.2 ± 1.0 (19) | 23.0 ± 2.9 (18) |
| ΔHexA(2S)-GalNAc(6S) | ND | ND | ND | ND | ND |
| ΔHexA-GalNAc(4S,6S) | 1.0 ± 0.2 (1) | 0.6 ± 0.2 (1) | 1.0 ± 0.3 (1) | 1.1 ± 0.1 (1) | 0.9 ± 0.4 (1) |
| Total CS disaccharides | 88.2 ± 3.5 (100) | 113.0 ± 3.3 (100) | 123.1 ± 11.5 (100) | 122.9 ± 1.6 (100) | 129.5 ± 6.9 (100) |
| Relative expression of *FAM20B* | 1.0 | 2.3 | 3.1 | 1.2 | 1.1 |
| Relative expression of *FAM20C* | 1.0 | 1.0 | 1.1 | 2.6 | 3.6 |

[a]The values are expressed as pmol of disaccharide per mg of dried homogenate and represent the mean ± s.d. (n = 3).

[b]ND, not detected.

[c]Quantitative real-time RT-PCR was used to measure relative amounts of the *FAM20B/Fam20b* or *FAM20C/Fam20c* transcripts. The respective mRNA levels were normalized to that of *Gapdh*.

Source data are provided as a Source Data file.

**Supplementary Table 5** Disaccharide composition of CS chains from stable clones of MC3T3-E1 cells.

| CS disaccharides | pmol $mg^{-1}$ (mol%)[a] | | | |
|---|---|---|---|---|
| | Parental | Mock | C6ST-1 OE #1 | C6ST-1 OE #2 |
| ΔHexA-GalNAc | 22.0 ± 3.0 (5) | 24.4 ± 1.4 (6) | 22.8 ± 1.0 (5) | 20.3 ± 2.2 (5) |
| ΔHexA-GalNAc(6S) | 7.3 ± 0.6 (2) | 7.2 ± 0.6 (2) | 11.0 ± 0.9 (3) | 16.4 ± 3.8 (4) |
| ΔHexA-GalNAc(4S) | 366.4 ± 24.0 (90) | 377.1 ± 22.5 (90) | 376.3 ± 2.8 (89) | 383.1 ± 18.8 (89) |
| ΔHexA(2S)-GalNAc(6S) | ND[b] | ND | ND | ND |
| ΔHexA-GalNAc(4S,6S) | 11.8 ± 1.2 (3) | 12.6 ± 1.3 (3) | 12.2 ± 1.4 (3) | 12.1 ± 1.2 (3) |
| Total CS disaccharides | 407.6 ± 28.6 (100) | 421.3 ± 24.5 (100) | 422.4 ± 2.9 (100) | 431.9 ± 20.4 (100) |
| Relative expression[c] | 1.0 | 1.0 | 1.8 | 2.7 |

[a]The values are expressed as pmol of disaccharide per mg of dried homogenate and represent the mean ± s.d. (n = 3).

[b]ND, not detected.

[c]Quantitative real-time RT-PCR was used to measure relative amounts of the *C6st1* transcript. *C6st1* mRNA levels were normalized to that of *Gapdh*.

Source data are provided as a Source Data file.

**Supplementary Table 6** Disaccharide composition of CS chains from BMSCs derived from wild-type or *C6ST1* transgenic mice.

| CS disaccharides | pmol $mg^{-1}$ (mol%)[a] | |
|---|---|---|
| | Wild-type | *C6ST1* transgenic |
| ΔHexA-GalNAc | 7.3 ± 2.0 (1) | 6.3 ± 2.7 (1) |
| ΔHexA-GalNAc(6S) | 14.3 ± 3.2 (2) | 49.5 ± 5.7 (6) |
| ΔHexA-GalNAc(4S) | 783.6 ± 31.9 (94) | 716.4 ± 58.3 (89) |
| ΔHexA(2S)-GalNAc(6S) | 2.5 ± 0.8 (0) | 8.0 ± 2.3 (1) |
| ΔHexA-GalNAc(4S,6S) | 29.5 ± 1.4 (4) | 22.2 ± 4.8 (3) |
| Total CS disaccharides | 837.2 ± 38.8 (100) | 802.4 ± 73.3 (100) |

[a]The values are expressed as pmol of disaccharide per mg of dried homogenate and represent the mean ± s.d. (n = 3).

Source data are provided as a Source Data file.

**Supplementary Table 7** Antibodies used in this study

| Antibody (clone) | Source of species | Cat. No. | Dilution etc. | Applications | Suppliers |
|---|---|---|---|---|---|
| Anti-FLAG (M2) | mouse | #F1804 | 1:1,000 | Western blotting | Sigma-Aldrich |
| Anti-His-tag | rabbit | #2365 | 1:1,000 | Western blotting | CST |
| Anti-FAM20C | rabbit | #25395-1-AP | 1:1,000 | Western blotting | Proteintech |
| | | | 1 µg/sample | Immunoprecipitation | |
| Anti-FAM20B (1018512) | mouse | #MAB8427 | 1:250 | Western blotting | R&D systems |
| Anti-C4ST-1 (L18) | mouse | #sc-100868 | 1:200 | Western blotting | Santa Cruz |
| Anti-GAPDH (5A12) | mouse | #014-25524 | 1:1,000 | Western blotting | Fujifilm Wako |
| Anti-N-cadherin (GC4) | mouse | #C3865 | 5 ng $ml^{-1}$ | Neutralization | Sigma-Aldrich |
| Anti-cadherin-11 (16G5) | mouse | #ab151446 | 5 ng $ml^{-1}$ | Neutralization | Abcam |
| Anti-ERK1/2 | rabbit | #9102 | 1:1,000 | Western blotting | CST |
| Anti-phospho-ERK1/2 | rabbit | #9101 | 1:1,000 | Western blotting | CST |
| Anti-Smad1 | rabbit | #9743 | 1:1,000 | Western blotting | CST |
| Anti-phospho-Smad1/5/8 | rabbit | #9511 | 1:1,000 | Western blotting | CST |
| Anti-Smad3 | rabbit | #9523 | 1:1,000 | Western blotting | CST |
| Anti-phospho-Smad3 | rabbit | #9520 | 1:1,000 | Western blotting | CST |
| Anti-mouse IgG HRP-linked | sheep | #NA931 | 1:5,000 | Western blotting | Cytiva |
| Anti-rabbit IgG HRP-linked | donkey | #NA934 | 1:10,000 | Western blotting | Cytiva |
| Anti-goat IgG HRP-linked | mouse | #sc-2354 | 1:5,000 | Western blotting | Santa Cruz |

**Supplementary Table 8** Primers used for construction of expression plasmids

| Construct | | Forward/sense (5'–3') | Reverse/antisense (5'–3') |
|---|---|---|---|
| pCMV-FAM20Cs | | CAAGATCT[a]GGCAGAACGCGCTCCAGG | CAAGATCTCCCCTCTGTCTCTGTCCC |
| pCMV-FAM20C (G379R) | IM[b] | CACGCCCTGTGCAGGAAGCCAGACC | GGTCTGGCTTCCTGCACAGGGCGTG |
| pCMV-FAM20C (G379E) | IM | CACGCCCTGTGCGAGAAGCCAGACC | GGTCTGGCTTCTCGCACAGGGCGTG |
| pCMV-FAM20C (L388R) | IM | TCGAGGGCTCGCGGGCGGCCTTCCT | AGGAAGGCCGCCCGCGAGCCCTCGA |
| pCMV-FAM20C (R549W) | IM | GCCCTGGACCGGTGGCTCCGCGTCGT | ACGACGCGGAGCCACCGGTCCAGGGC |
| pEF-BOS/IP-FAM20B | | CGGGATCCTCAGCTGCCAACCGGGAGGAC | CGGGATCCACTTCCAATCCATCTCATACC |
| pEF-BOS/IP-FAM20B (D309G) | IM | GCTCATCCTTCTTGGTAATGCCAAAAGCTT | AAGCTTTTGGCATTACCAAGAAGGATGAGC |
| pEF-BOS/IP-FAM20Cs | | CAAGATCTGAGCCCGGCTGTTCGTG | CAAGATCTCCCCTCTGTCTCTGTCCC |
| pEF-BOS/IP-FAM20C (D478G) | IM | TTCATCATCCACTTAGGCAATGGAAGAGGG | CCCTCTTCCATTGCCTAAGTGGATGATGAA |
| p3XFLAG-CMV-8-FAM20B | | GCTCTAGATCAGCTGCCAACCGGGAGGAC | GCTCTAGAACTTCCAATCCATCTCATACC |
| p3XFLAG-CMV-8-FAM20Cs | | GCTCTAGAGAGCCCGGCTGTTCGTG | GCTCTAGACCCCTCTGTCTCTGTCCC |
| pcDNA3Ins-His-C4ST-1 | | GCTCTAGACTGCAGGAACTCTACAAC | CGGGATCCTAAAAAGCATGATTCTCTC |
| pEF-BOS/IP-XylT-1 | | GAAGATCTGACAGCAACAACGAGAACTCTG | GAAGATCTCTTTCCCGTTGAGATCCTGCTG |
| pEF-BOS/IP-GalT-I | | CGGGATCCTCTGGGGACGTGGCCCGG | CGGGATCCTCAGCTGAATGTGCACCA |
| pEF-BOS/IP-GalT-II | | CCGGATCCGAGCCCGGGGACCCCAGG | CGGGATCCTCAGGGGATGCCCTCCCTTCT |

[a]The underlines indicate restriction enzyme sites.

[b]IM, internal mutagenic primers.

**Supplementary Table 9** Primers used for quantitative real-time RT-PCR.

| Human gene | Forward (5'–3') | Reverse (5'–3') |
|---|---|---|
| *GAPDH* | ATGGGTGTGAACCATGAGAAGTA | GGCAGTGATGGCATGGAC |
| *FAM20B*[a] | GCCTTAAAATCTGCCATGGC | GGCATCCTGTCTTCCACCAG |
| *FAM20C*[a] | GAGAGGCACAATGCGGAGAT | GGATCTCCTTGGTCATGTTG |
| *CSGALNACT1* | AGAAGAAATAAATGAAGTCAAAGGAATAC | GAAGTAGATGTCCACATCACAG |
| *CSGALNACT2* | CCTAGAATCTGTCACCAGT | GTTAAGGAATTCGGCTGAGAAATA |
| *C4ST1* | AAACGCCAGCGGAAGAA | GGGATGGCAGAGTGAGTAGA |
| *C4CT2* | GAGGGAAAGTTCTTTGTTTAAGTG | CGGCCTTAACAGCCATAAT |
| *C6ST1* | TCTGCCATTGGCTTGAAC | CATGCAGACATGAAATAGCAAAC |
| Mouse gene | Forward (5'–3') | Reverse (5'–3') |
| *Gapdh* | CATCTGAGGGCCCACTG | GAGGCCATGTAGGCCATGA |
| *C6st1* | CTGGCATTTGTGGTCATAGTTT | AAGAGAGATGCATTCTCCGATAAG |
| *Akp2* | CCTGACTGACCCTTCGC | GTCAAGGTGTCTTTCTGGGA |

[a]These primer sets are applicable for quantitative real-time RT-PCR of the respective mouse counterparts.
